# The Exposome – a New Approach for Risk Assessment

**DOI:** 10.14573/altex.2001051

**Published:** 2020

**Authors:** Fenna C. M. Sillé, Spyros Karakitsios, Andre Kleensang, Kirsten Koehler, Alexandra Maertens, Gary W. Miller, Carsten Prasse, Lesliam Quiros-Alcala, Gurumurthy Ramachandran, Stephen M. Rappaport, Ana M. Rule, Denis Sarigiannis, Lena Smirnova, Thomas Hartung

**Affiliations:** 1 Johns Hopkins University, Bloomberg School of Public Health and Whiting School of Engineering, Environmental Health and Engineering, Baltimore, MD, USA;; 2 Aristotle University of Thessaloniki, Center for Interdisciplinary Research and Innovation, HERACLES Research Center on the Exposome and Health, Thessaloniki, Greece;; 3 Columbia University Mailman School of Public Health, New York, NY, USA;; 4 University of California, School of Public Health, Berkeley, CA, USA;; 5 University School for Advanced Study (IUSS), Science, Technology and Society Department, Environmental Health Engineering, Pavia, Italy;; 6 University of Konstanz, Konstanz, Germany

## Abstract

Complementing the human genome with an exposome reflects the increasingly obvious impact of environmental exposure, which far exceeds the role of genetics, on human health. Considering the complexity of exposures and, in addition, the reactions of the body to exposures – i.e., the exposome – reverses classical exposure science where the precise measurement of single or few exposures is associated with specific health or environmental effects. The complete description of an individual’s exposome is impossible; even less so is that of a population. We can, however, cast a wider net by foregoing some rigor in assessment and compensating with the statistical power of rich datasets. The advent of omics technologies enables a relatively cheap, high-content description of the biological effects of substances, especially in tissues and biofluids. They can be combined with many other rich data-streams, creating big data of exposure and effect. Computational methods increasingly allow data integration, discerning the signal from the noise and formulating hypotheses of exposure-effect relationships. These can be followed up in a targeted way.

With a better exposure element in the risk equation, exposomics – new kid on the block of risk assessment – promises to identify novel exposure (interactions) and health/environment effect associations. This may also create opportunities to prioritize the more relevant chemicals for risk assessment, thereby lowering the burden on hazard assessment in an exposure-driven approach. Technological developments and synergies between approaches, quality assurance (ultimately as Good Exposome Practices), and the integration of mechanistic thinking will advance this approach.

“Progress is impossible without change, and those who cannot change their minds cannot change anything.”

George Bernard Shaw (1856-1950)

“If you change the way you look at things, the things you look at change.”

Wayne Dyer (1940-2015)

## 1 Introduction

Claiming a new ’ome has become fashionable in the post-human genome era. Monya Baker (2013), in her piece in *Nature*, claimed the number to be in the thousands. She wrote: “*Botanist Hans Winkler had no idea what he was starting back in 1920, when he proposed the term ‘genome’ to refer to a set of chromosomes. Other ’omes existed even then, such as biome (collection of living things) and rhizome (system of roots), many of them based on the Greek suffix ‘-ome’ – meaning, roughly, ‘having the nature of.’ But it was the glamorization of ‘genome’ by megabuck initiatives such as the Human Genome Project that really set the trend in motion, says Alexa McCray, a linguist and medical informatician at Harvard Medical School in Boston, Massachusetts*. ‘*By virtue of that suffix, you are saying that you are part of a brand-new exciting science*.’” (Baker, 2013). The human toxome, claimed earlier in this series of articles (Hartung and McBride, 2011), and subsequently an NIH Transformative Research Grant (Bouhifd et al., 2015a), was one of Baker’s examples addressed in a favorable way while poking fun at others. She concluded: “*Ideally, branding an area as an ’ome helps to encourage big ideas, define research questions and inspire analytical approaches to tackle them*.”

Christian Wild in 2005 possibly made the wi(l)dest claim by suggesting the “exposome.” He even could have called it the “lifeome” as it encompasses every impact of any exposure on the human body, e.g., chemicals, infections, radiation, as well as internal modulating factors such as the microbiome and metabolism. The goal of the exposome was to comprehensively measure these imprints by transcriptomics, epigenomics, proteomics, metabolomics, and others, primarily in biofluids such as blood and urine. The exposome can include well-established measures of exposure, such as traditional biomonitoring or environmental monitoring, but also includes untargeted discovery of unknown chemicals of biological importance. The exposome concept thus refers to the totality of environmental exposures from conception on, including external and internal components. Expanding on Wild (2005), Rappaport and Smith (2010) functionalized the exposome as the totality of chemicals that can be measured in blood, and in 2014, Rappaport defined the “blood exposome” (Rappaport et al., 2014). The field was furthered in a number of seminal articles (Vineis et al., 2009; Wild, 2009; Miller and Jones, 2014) and books (Miller, 2013; Dagnino and Macherone, 2019). This concept calls to mind a quote from Vietnamese Buddhist monk and peace activist Thich Nhat Hanh: “*You carry Mother Earth within you. She is not outside of you. Mother Earth is not just your environment*.”

The paper by Rappaport and Smith (2010) was critical in popularizing the exposome concept, which was furthered in a National Academies of Sciences report (NRC, 2010) and the National Institute of Environmental Health Sciences (NIEHS) Exposome Workshop in January 2015 (Dennis et al., 2017). Most of the discussion about an exposome, and most of this article, centers on human exposures and health effects. However, the concept can similarly be applied to environmental perturbations. In 2012, the National Research Council (NRC) of the US National Academy of Sciences defined the “eco-exposome” as “*the extension of exposure science from the point of contact between a stressor and receptor inward into the organism and outward to the general environment, including the ecosphere*” (NRC, 2012).

This article was very much inspired by the creation of the Johns Hopkins Exposome Collaborative^1^ and its launch event on November 8, 2019. Members of the organization and guest speakers at the event were invited to collaborate on this piece.

## 2 Nature or nurture? – The debate has a new tool

“*The exposome captures the essence of nurture; it is the summation and integration of external forces acting upon our genome throughout our lifespan*” (Miller, 2013; Miller and Jones, 2014). Genetics can only explain a minor part of human diseases (Welter et al., 2014; Rappaport, 2016; Jones, 2016). Craig Venter, shortly after the completion of the human genome sequence, stated: “*We simply do not have enough genes for this idea of biological determinism to be right*” (McKie, 2001). After more than 2,000 genome-wide association studies (GWAS), relatively little disease variation has been explained (Visscher et al., 2012), and the median attributable risk of 28 diseases across pairs of monozygotic twins was found to be only 16% – indicating a rather small influence of entire genomes on disease risk (Rappaport, 2016). These results are consistent with the estimate that 70 to 90% of disease risks are environmental factors (Willett, 2002). This pre-dominance of exposure in causing disease is not reflected in current research (Rappaport, 2016): For example, in PubMed citations (Feb. 6, 2016), “disease causes AND genetics” resulted in 566,685 hits, whereas “disease causes AND exposure” resulted in only 71,922. Miller (2013) summarized: “*It is odd that the majority of the discussion of Darwin occurs among geneticists when natural selection is primarily driven by the environment. The environment, viz., the exposome, is what is driving natural selection… if one removes the external forces acting upon our genome, evolution would grind to a halt*.” While less emphasized in research, public opinion is actually characterized by chemophobia, as aptly expressed by actress Sandra Bullock: “*If you can’t pronounce it, you probably shouldn’t be putting it in your body or in your environment*.”^2^ Most people, of course, would have problems pronouncing the names of some vitamins and nutrients. Others, such as former vice president of the United States Dan Quayle (1947-), opine: “*It isn’t pollution that’s harming the environment. It’s the impurities in our air and water that are doing it*.”^3^ Sarcasm or ignorance? Who can tell?

The increasingly popular Developmental Origins of Health and Disease (DOHaD) hypothesis (Gluckmann and Hanson, 2006) suggests that exposures during critical and sensitive windows of susceptibility may induce epigenetic changes to prepare the embryo or fetus for extrauterine life; this stresses the relevance of the intrauterine exposome. Increasingly, a “life course” approach to understanding chronic diseases (Ben-Shlomo and Kuh, 2002) is being embraced. This indicates a strong impetus for exposure sciences. As Miller and Jones (2014) succinctly stated, “*We need an output of environmental exposures as tangible as the mutated gene*.” New approaches to identify environmental causes of diseases are fundamental to improving individual and population health (Baccarelli, 2019). Traditionally, this was attempted one chemical, one hazard/disease, and one population at the time and could take up to 50 years, even where there was clear exposure and strong correlation of disease such as smoking and lung cancer. This approach misses all the interactions of mixtures, many confounders (individual differences, including disease history of individuals), and cannot typically be integrated easily with mechanistic understanding. Sure, epidemiologists have developed tools to identify covariables, but in each case only a *few likely* ones and plausible mechanisms of action are part of the Bradford-Hill criteria (Hartung et al., 2013) as supportive elements for causation.

Instead of solving all of these associations one at the time, the exposome throws everything on one pile – first for the individual and then for the population. Each and every piece is incomplete: Who recalls the Sunday in 1986, when they sprayed a pesticide in the garden? The exposome reduces the pile, on the one hand, to what is measurable today, either the contaminant or the imprint it left. This reminds us somewhat of diagnosis in Traditional Chinese Medicine, focusing on pulse and tongue only, upon which relevant diseases are assumed to have an impact. This comparison is not meant to belittle this diagnostic approach, where a sophisticated pulse assessment takes up to 30 minutes (for a nice summary, see^4^), but to contrast to the Western medicine approach of measuring everything. Similarly, exposomics focuses on what can be measured in biofluids (blood or urine). This is still a lot, given the breadth of modern omics technologies.

## 3 The exposome – more than chemical exposures

**Fig. 1:**
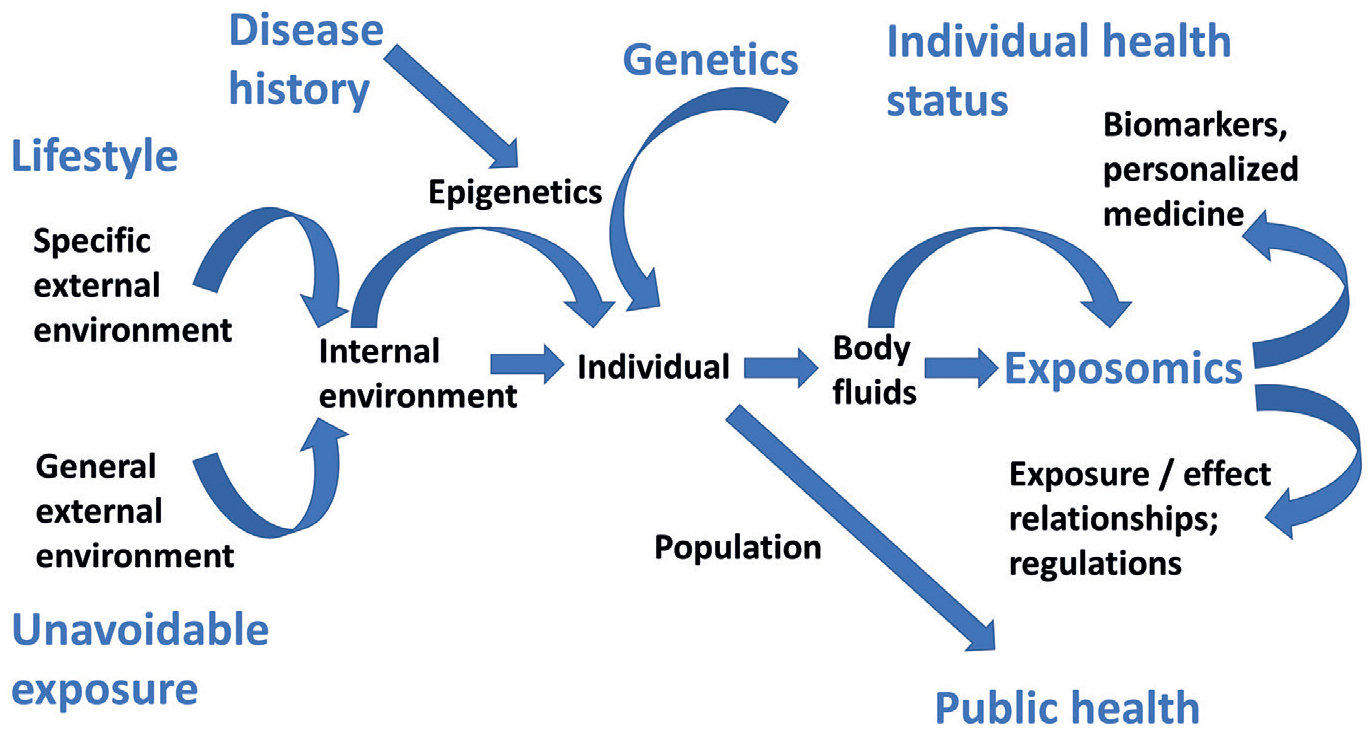
The human exposome concept The health status of an individual is imprinted by the specific external (lifestyle) and general external (unavoidable) environment together with the internal environment they influence. Through epigenetics, the individual’s disease history as well as directly and indirectly genetics further impact, determining the exposome and reflecting the person’s health status. On a population level, exposure/effect relationships can be deduced, supporting regulations to effect public health.

When considering exposures, we typically think of environmental chemicals. The American comedy writer Robert Orben (1927-) pointedly said: “*There’s so much pollution in the air now that if it weren’t for our lungs there’d be no place to put it all*.”^5^ However, there is far more to consider about exposure. We are not 70 kg rats (Hartung, 2009) – already because approximately 33% of adults and 17% of children in the United States are currently estimated to be obese (Ogden et al., 2012), and 70 kg is a somewhat outdated average for the weight of a human. Other lifestyle (specific external) factors like smoking, diet, and recreational activities, may also play a role in human health and the exposome. Among the general external factors that we as individuals have little influence on are the (residential) environment, climate, socio-economic status and its consequences, as well as many others (summarized in Fig. 1).

Miller and Jones (2014) stressed “*The exposome must explicitly include how our bodies respond to environment pressures, including epigenetic changes and mutations, as well as the complex chemistry resulting from the biochemical reactions that sustain our lives*.” This prompted their redefinition (which we adopt for the purpose of this article; for other definitions see the glossary):

*Exposome:* The cumulative measure of environmental influences and associated biological responses throughout the lifespan, including exposures from the environment, diet, behavior, and endogenous processes.

They see three distinct differences in this definition from Wild’s original definition:

1. The concept of the cumulative biological responses (ongoing adaptations and maladaptations to external forces and chemicals), i.e., the body’s response to these challenges.
2. The inclusion of behavior (in a very broad context, to include personal and volitional actions and those that result from family, community, or social units going beyond lifestyle to include the dynamic interaction with our surroundings, relationships, interactions, and physical and emotional stressors).
3. The addition of “endogenous processes” (i.e., the body’s complex biochemical reactions)

This definition therefore recognizes the need to consider the totality of external and internal factors.

Beside the omics of biofluids, the exposome makes use of biomonitoring and biomarker data from omics or other techniques (internal), sensors for environmental or personal monitoring (specific external), geographic information systems (GIS) (general external), conventional methods such as survey instruments or job-exposure matrices (specific external), and reality mining from social networks or other sources (general external) (DeBord et al., 2016). The rapid development of material sciences and electronic technologies has enhanced our ability to measure and map specific exposures: These include, for example, sensors that measure a wide range of targeted environmental pollutants, monitors for physiological parameters (i.e., blood pressure, heart rate, stress, physical activity, sleep patterns), GPS devices for monitoring location, mobile phones for dietary assessments and social interactions, and high resolution atmospheric models for predicting ground-level pollutant levels as well as high resolution mass spectrometry and deep sequencing for adducts and metabolic and epigenetic imprints (Niedzwiecki et al., 2019).

## 4 The exposome – an unattainable vision?

One key technology for assessing the exposome is metabolomics, because it is the omics technology closest to a phenotype. Genes are not necessarily transcribed, mRNAs are not always translated, and some proteins are either not processed or not reflective of the phenotype. When metabolites change, the phenotype changes. Mass-spectroscopy-based metabolomics is the key technology for characterizing chemical exposures based on their remnants and metabolites in the body. It should not take a historian (David Christian, 1946-) to explain the obvious: “*Living organisms are created by chemistry. We are huge packages of chemicals*.” However, given the inherent totality of an ’ome, even the metabolome falls short due to lost coverage of small molecules owing to factors such as sample preparation, choice of chromatography, ionization, measurement size window, etc. Consequently, only a fraction of the chemicals present in a sample is measured. The goal of exposomics, therefore, is to cast a wide net when measuring classes of chemicals to form hypotheses regarding putative biomarkers for follow-up.

Rappaport, in his foreword to Dagnino and Macherone (2019), wrote: “*The vast chemical space and dynamic range of the blood exposome emphasize the importance of untargeted methods for discovering causes of disease. In fact, state-of-the-art liquid chromatography-mass spectrometry can now detect thousands of small molecules and proteins in a few microliters of archived blood from prospective cohorts. Likewise, preprocessing methods for metabolomics and proteomics are rapidly evolving to filter and normalize untargeted proteomics and metabolomics data that are appropriate for statistical analyses. Thus we can now conduct exposome-wide association studies (EWAS) as complements to GWAS in epidemiologic studies (*Rappaport, 2012*) and thereby identify causal ‘signatures and fingerprints’ of exposure that fueled Wild’s speculation in 2005*.” Keeping in line with the pathway perturbation concept, not all perturbations at the level of gene expression should be taken into account, because many would result in adaptive, not toxic, responses (Smirnova et al., 2015). A reliable way to identify the perturbations that lead to toxic responses is to couple gene expression-based molecular response pathways with the prevalent pathways identified from bioinformatic analysis of metabolite profiles. Metabolomics data provide a closer link to potential phenotypic/clinical observations. Joint transcriptomic and metabolomic data analysis allow us to identify, among the perturbed pathways, those more likely to be associated with adverse outcomes (Hartung and McBride, 2011; Sarigiannis et al., 2018a). Thus, the main weakness of transcriptomics, namely, the failure to distinguish between adaptive and toxic responses, can be overcome. Metabolomics is developing rapidly and is increasingly entering toxicology (Bouhifd et al., 2013; Ramirez et al., 2013), including *in vitro* toxicology (Ramirez et al., 2018). This massive rise in the coverage of large and small molecules in a given sample increases the demand for bioinformatics and cheminformatics from data processing tools to comprehensive databases, and statistical and computational tools for modeling metabolic networks, with the inherent pitfalls illustrated previously: We showed on the one hand that using a data analysis approach based exclusively on pathway annotations has the potential to miss much that is of interest; in the case of misidentified metabolites, it can produce perturbed pathways that are statistically significant yet uninformative for the biological sample at hand. On the other hand, a targeted approach, by narrowing focus and minimizing – but not eliminating – misidentifications, renders the likelihood of a spurious pathway much smaller. The limited number of metabolites, however, also makes statistical significance harder to achieve (Maertens et al., 2017).

With so much information being amassed, new types of metabolomic databases are emerging (Go, 2010) that are not only storing, managing, and analyzing metabolomic data, but also serve as “*gateways to the vast information space of metabolism in living systems*.” The mushrooming of metabolomics has driven the development of a number of freely and commercially available databases that provide information on the chemical structures, physicochemical and pharmacological properties, spectral profiles, experimental workflows, and biological functions of metabolites. These comprehensive, well-annotated, and user-friendly databases (Go, 2010) are able to store and manage the vast amounts of disparate and complex data with relevant associated annotations and feature user-friendly interfaces with a range of tools that can be tailored according to a specific project. However, we are still missing the equivalent of a gene ontology^6^, i.e., a metabolite ontology connecting each metabolite to its biological role. In short, a great deal of data infrastructure is still needed before metabolomics can begin to compete with transcriptomics. On the other hand, the lessons learned from transcriptomics about the importance of publicly available datasets, data curation, and ontologies can help point the way.

## 5 Epigenetics as the bridge between genetics and the environment is key to exposomics

Epigenetics arbitrates between genes and the environment. Epigenetic mechanisms control spatial and temporal transcriptomic activity. They include (1) DNA methylation, (2) nine types of histone modifications (acetylation, methylation of lysines, methylation of arginines, phosphorylation, ubiquitylation, sumoylation, ADP ribosylation, deamination and proline isomerization), (3) epitranscriptome (100+ chemically distinct modifications of ribonucleosides), and (4) non-coding RNAs (microRNAs (miRNAs) and long intervening noncoding RNAs (lincRNAs)) that regulate cell-specific gene expression by influencing DNA-protein interactions and chromatin structure (Torres-Padilla et al., 2007; Mikkelsen et al., 2007; van der Heijden et al., 2008; Lee et al., 2012; Smith et al., 2012; Cantone and Fisher, 2013). Dynamic epigenetic programming is regulated during early development; methylation patterns, for example, are then mitotically inherited in somatic cells, thus preserving cell identity of differentiated cells (Smith et al., 2012; Li et al., 2013; Cantone and Fisher, 2013). Emerging data suggest non-traditional roles for CpG and non-CpG (i.e., CpT, CpA, or CpC) methylation (Ziller et al., 2011) that may play a particularly important role in the response to the environment. In addition, disruption of imprinted genes’ tissue-specific expression patterns can lead to a variety of behavioral phenotypes (reviewed in Plasschaert and Bartolomei, 2014). Epigenetic regulation is hereditable but can be influenced by environmental stimuli, *in utero* circumstances, and aging. As botanist Luther Burbank (1849–1926) noted, “*Heredity is nothing but stored environment*.”^7^ How true for the exposome and epigenetics!

Goodman (2017) recently discussed epigenetics in toxicology, noting that toxicologists have embraced epigenetic regulation of gene expression, as evidenced by their increasing focus on discerning potential epigenetic mechanisms by which chemical and physical agents might cause toxicity. There appears to be a relation between the epigenome and disease (Jirtle and Skinner, 2007; Waterland and Michels, 2007), and epigenome-wide association studies (Rakyan et al., 2011) are being carried out. Smirnova et al. (2012) summarize epigenetics in a broad variety of toxicological studies. There is, however, no consensus about how this might be systematically incorporated into risk assessment.

Because they can be dynamically regulated while being heritable, epigenetic marks serve at the interface of genes and the environment and have become likely candidates for a mechanistic link between exposure and disease susceptibility. In one of our previous articles in this series, we introduced the term “epigenetic scar” or memory (Smirnova et al., 2015), defined as “*long-term changes in epigenetics induced by stressors, which affect regulation of gene expression*.” This imprinting can lead to disease manifestations, but also can be protective by leveraging the homeostasis of the cell, tissue, organ or organism in response to changes in the exposome. An important aspect of the robustness of an organism’s epigenetic response to exposure is redundancy.

In particular, miRNAs have some interesting properties that predestine them as biomarkers (Smirnova and Maertens, 2019):

- miRNA genes are very often encoded on several chromosomes.
- One miRNA may regulate hundreds of mRNAs and one mRNA can be regulated by several miRNAs.
- Often, one mRNA has several binding sites for different miRNAs.
- 60% of mRNAs are miRNA targets.
- A relatively small number of miRNAs, e.g., *Homo sapiens*: ~2578
- Can be measured sensitively by PCR amplification
- Conserved through the phyla, i.e., easy to translate between species
- Fine-tuning of mRNA network through positive and negative feedback loops
- miRNA-targets more likely to be affected by chemicals than non-miRNA-targets
- miRNAs are more stable than mRNAs, allowing analysis in archived formalin-fixed paraffin-embedded (FFPE) blocks, biofluids, archived and degraded samples.
- Circulating (secreted) miRNAs in biofluids lend themselves as biomarkers for organ toxicity and injury.
- Organ and developmental stage-specific expression eases interpretation.

From one perspective, epigenetic mechanisms appear to buffer “noisy” gene expression to maintain homeostasis in response to environmental stress. From another, epigenetic marks can be inherent targets of the exposure. Potential mechanisms for environment-induced DNA methylation changes include increased/decreased expression or inhibition of DNA methyltransferases (Duncan et al., 2013; Schaevitz et al., 2014) and toxicant-induced reactive oxygen species leading to demethylation of DNA (Coulter et al., 2013). Changes in the exposome may alter miRNA expression, which may change the fine-tuning of gene expression and lead to adverse outcomes in the long run. As elegantly shown by Fraga et al. (2005), differences in the epigenetic signatures of monozygotic twins were indistinguishable at the age of three, and drifted apart later in life, suggesting exposure contribution to the epigenome. Another study demonstrated influences of the environment on the epigenome using the yellow agouti (*Avy*) mouse model, in which coat color variations depend on epigenetic marks established early in development; this model has been used to investigate the impacts of exposure on the fetal epigenome (Feil and Fraga, 2011).

Plusquin et al. (2019) note that “*Genomic DNA isolated from blood cells or other pertinent tissues is being expansively exploited for the discovery of biomarkers of effect and exposure. Technology to measure epigenetic marks on a genomic scale complemented with novel tools for data-analysis have recently been developed and continue to be enhanced*.” Increasingly, they are therefore included in exposomics studies – especially miRNAs as biomarkers for environmental exposure (recently systematically reviewed by Vrijens et al., 2015). In any case, addressing the epigenetic response to perturbations in the exposome should be a key to biomarker identification.

## 6 Exposomics gives a spin to traditional exposure biomonitoring, overcoming some of its limitations

Biomonitoring, in the closer sense of chemicals (as discussed in NRC, 2006), involves the quantitative measurement of the accumulation of chemicals in tissues:

*Biomonitoring:* The measurement of the body burden of toxic chemical compounds, elements, or their metabolites, in biological substances.

Recently, with the continuous improvement of measurement technologies, such studies have grown in number of studies, number of chemicals measured, and number of people monitored. This is met with some skepticism, as our ability to measure expands faster than our ability to interpret, and again, in contrast, emphasizes the hypothesis-generating focus of exposomics. For example, Juberg et al. (2008) noted that “*Biomonitoring reveals the amount of a chemical in an individual’s body, but such knowledge is largely meaningless unless we know at what level in body fluids or tissues health consequences do and don’t occur*.” Similarly, the National Academy of Sciences stated: “*The ability to generate new biomonitoring data often exceeds the ability to evaluate whether and how a chemical measured in an individual or population may cause a health risk or to evaluate its sources and pathways for exposure*.” Clearly, exposomic investigations must be followed up with specific hypotheses to provide the mechanistic understanding linking biomonitoring data with disease pathways and morbidity.

The exposome offers new opportunities for interpreting biomonitoring data as shown by Pino et al. (2017). Still, biomarkers have some disadvantages, as noted by Náray and Kudász^8^ of the Hungarian Institute of Occupational Health (in their case for monitoring workers). They note that biomarkers:

- “are usually unable to specify the source of the exposure (occupational or non-occupational);
- may not be sufficiently specific to a particular chemical;
- are not suitable for identification of workplace contaminations in general;
- may be interfered by other chemicals in the biological medium (e.g. medications);
- are not useful at all for the assessment/monitoring of acute and/or local toxic effects (e.g. irritation);
- *and the provision of samples for biomonitoring may be a burden for workers (e.g. blood samples)*.”

This shows that a specific use area for biomonitoring or exposomics might come with specific additional considerations. Exposomics contrasts with traditional biomonitoring, which is typically limited to a select group of known chemicals (usually in the tens and rarely lower hundreds) (Buekers et al., 2018). Some prominent disadvantages are that such studies (e.g., the National Health and Nutrition Examination Survey, NHANES^9^) do not take continuous repeated measures over time, thereby limiting detection of short-lived chemicals and suspected chemicals of concern, which are less likely to be captured (Dennis et al., 2017). The focus is usually on the relatively exact measurement of individual chemicals, which is time-intensive for development and validation, and expensive and time-consuming for execution, as well as often limited to few laboratories with such capabilities. Multiple methods are required for a large suite of chemicals, and typically 0.5–2 mL of blood or other biospecimens are needed per analyzed chemical. Additionally, chemicals added for monitoring are not always the most important ones from a toxicologic perspective.

Biomonitoring increasingly incorporates biomarker measurement. The concepts of exposome and biomarkers overlap closely (Rappaport, 2012).

*Biomarker:* A characteristic that can be objectively measured and evaluated as an indicator of normal biological processes, pathogenic processes or pharmacological responses to a therapeutic intervention

(Puntmann, 2009)

Biomarker discovery has grown dramatically in the quest for biomarkers of disease in drug development and diagnostics. Rappaport (2012) distinguishes between biomarkers of environmental exposure (causal pathway) and biomarkers of disease (reactive pathway). Compared to biomonitoring, which assesses the presence of a chemical, biomarkers have a proven predictive value. While biomonitoring would measure a given chemical in any tissue or body fluid, a biomarker would be one that correlates with the extent of exposure or its effect (though by including more biomarkers, the difference becomes increasingly blurred). The World Health Organization (WHO) defined in 1993^10^: “‘*Biomarkers*’*, are recognized as providing data linking exposure to a chemical with internal dose and outcome and as relevant to the process of risk assessment*.” Over the last decades, the validation and acceptance of biomarkers has been formalized (Goodsaid and Mattes, 2013). The FDA-NIH Biomarker Working Group has made the BEST (Biomarkers, EndpointS, and other Tools) Resource available^11^ for further reference.

Notably, the biomarker concept also has been applied to *in vitro* research (Blaauboer et al., 2012), in part with the hope that biomarkers identified to reflect a mechanism might translate to biomarkers in humans. This may especially hold true for extracellular (secreted or by cell lysis) substances that might also be found in biofluids.

Exposomics uses omics and other data sources to discover biomarkers of both exposure and disease by casting a wide net and accepting that many biomarkers may actually be patterns of signals instead of single compounds. The goal is ultimately not to measure a myriad of signals in each individual (patient), but to reduce the information to a biomarker equivalent. The mechanistic foundation of the biomarker concept as a “marker of biology” is advantageous and an important outcome of the data-driven exposome approach for chemical risk assessment (Steckling et al., 2018).

An additional advantage of the exposome concept is the integration of toxicokinetics for linking exposure (and exposure biomarkers) with internal dosimetry. Starting from exposure biomarker data (Sarigiannis et al., 2016), we are able to reconstruct external exposure, estimate the internal dose in the target tissue that results in the observed biological perturbation, and compare this to a relevant *in vitro* identified biological pathway activating dose (BPAD) (Judson et al., 2011).

## 7 Advantages of exposomics over other approaches of exposure assessment

The comprehensive coverage of various exposures over time and in combinations, as well as the diversity of individuals considered, is the key strength (and weakness) of the approach. Beyond this, the already quoted NIEHS workshop (Dennis et al., 2017) saw the following advantages of an exposome approach:

- Agnostic approaches are encouraged for detection of emerging exposures of concern.
- Techniques (and development of techniques) promote identification of unknown/emerging exposures of concern.
- Links exogenous exposures to internal biochemical perturbations
- A large number of features can be detected (> 10,000) for the cost of a single traditional biomonitoring analysis.
- Includes biomolecular reaction products (e.g., protein adducts, DNA adducts) for which traditional biomonitoring measurements are often lacking or cumbersome
- Requires a small amount of biologic specimen (~100 μL or less) for full-suite analysis
- Enables detection of “features” that are linked to exposure or disease for further confirmation
- Encourages techniques to capture short-lived chemicals
- Aims to measure biologically meaningful lifetime exposures, both exogenous and endogenous, of health relevance.

## 8 Challenges for exposomics

Exposome studies also come with their own set of challenges. Some see the lack of stringent *a priori* hypotheses, the multiple testing problem, and the resulting probability of false-positive findings as problems reflecting the traditional view in epidemiology (Wacholder et al., 2004) that the lack of a highly curated *a priori* hypothesis impacts robustness of findings (as they depend not only on the strength of the observed data, but also on the strength of the *a priori* hypothesis). This view, however, misses the hypothesis-generating character of the exposome. The goal is to select from among the myriad variables those worthy of follow-up. Dealing with multiple testing already at this stage is problematic.

### The challenge of exposure variability

The variability of exposure data (e.g., exposures with short biological half-lives and little constancy in the underlying exposure behavior) creates obvious difficulties in interpretation. While the genome is essentially static, the exposome varies throughout life, presenting challenges for epidemiologic studies due to temporal, spatial, and genetic variability in both exposures and individuals’ responses to the exposures. The variability in biological distribution of chemicals in the body (the toxicokinetic variability), i.e., how chemicals are distributed and metabolized differently by individuals (Tsaioun et al., 2016), impacts internal exposure studies. It is also important to know whether a single sample obtained at a given life stage represents the relevant exposure over time or if exposures during a critical window are more important. Even when exposome studies include lifetime exposures, they often do not place enough emphasis on defining and measuring windows of susceptibility (e.g., *in utero*) to accurately capture the most biologically important exposures. Here, it should be noted that the HEALS project in the EU has identified ten critical windows of susceptibility during the human life course, providing a comprehensive guide to designing future exposome studies properly.

### The measurement challenge

In 2014, Rappaport defined the “blood exposome” (Rappaport et al., 2014), showing that chemicals derived from foods, drugs, and endogenous sources covered the same dynamic range of blood concentrations (pM-mM), while those from pollution sources were present at levels three orders of magnitude lower (fM-μM). The ability to identify and quantify low-abundance analytes – most environmental chemicals – is still immature. We will thus often miss chemicals that are present at low levels. Depending on the methodology used, we cannot detect all analytes present (e.g., we can only measure chemicals that are isolated in a given extraction process), and may not be able to define a reference or baseline value. Issues of sample availability and quality, identification of unknown analytes, and capture of non-persistent chemicals remain challenges and must continue to be addressed. Each method also comes with its analytical measurement error. Importantly, biomarkers have not been defined for all environmental exposures (Steckling et al., 2018). Untargeted (feature) measurement promises the discovery of such biomarker (patterns), but also emphasizes the challenges of feature identification, quantification, and reproducibility.

### The challenge of method integration

The more exposures are included, the more measurement technologies and types of data have to be combined. This has financial, technical, expertise, and biostatistical consequences. For example, not all technologies can be applied to all subjects (e.g., small children, frail elderly subjects, and permissive/non-permissive work environments). Some measurements are too costly to be applied to large cohort studies. Very few investigators have expertise in all relevant measurement technologies, especially vis-a-vis quality assurance, and thus will require collaborations to increase coverage of the exposome in a given population. Another challenge is the ability to share and work on large omics datasets from different originating laboratories. For this purpose, the Human Toxome Project developed the Human Toxome Collaboratorium (Fasani et al., 2016), a cloud-based platform for data storage with metadata analyses and their documentation. This might be a path toward exposome workflows and reporting. The HEALS project in the EU developed the HEALS geo-database (HEALS GeoDB) as a repository of all geo-referenced measurements and data emanating from the HEALS techniques and methodologies.

### The challenge of statistical assessment of increasingly complex data sets

Big data refers not only to the volume of data, but also to their variety and the velocity of data accrual – the so-called 3Vs that are characteristics of big data. This stretches the limits of our existing analytical methodologies. “*Big data is transforming the traditional ways of handling data to make sense of the world from which it is collected. Statisticians, for instance, are used to developing methods for analysis of data collected for a specific purpose in a planned way. Sample surveys and design of experiments are typical examples. Big data, in contrast, refers to massive amounts of very high dimensional and even unstructured data which are continuously produced and stored with much cheaper cost than they are [sic] used to be. High dimensionality combined with large sample size creates unprecedented issues such as heavy computational cost and algorithmic instability*” (Pyne et al., 2016). Typically, big data originate from combining data from multiple sources using different technologies over time. Unavoidably, this introduces heterogeneity, experimental variations, and statistical biases that require more adaptive and robust procedures for analysis and curation (Megahed and Jones-Farmer, 2015; Wang et al., 2016; Saidulu and Sasikala, 2017; Reid, 2018^12^; Cox et al., 2018) that increasingly rely on machine learning (also termed artificial intelligence (AI)) (Di Ciaccio et al., 2012; Pyne et al., 2016; Hartung, 2018), also for environmental health (Sarigiannis et al., 2009).

### Database challenges

The application of untargeted metabolomics to identify environmental exposures correlated with human health has its own unique challenges (Dennis et al., 2017). The metabolomic aspect of analyses relies heavily upon library searching of spectra for annotation with standard confirmation coming later, which can be quite time-consuming and labor-intensive. The largest reference databases for metabolomics are the Metabolite and Tandem MS Database (METLIN)^13^ and the Human Metabolome Database (HMDB)^14^. To date, METLIN and HMDB have largely focused on naturally occurring metabolites. According to Dennis et al. (2017), the number of compounds in METLIN and HMDB that may be potentially relevant to exposure studies has not yet been carefully assessed. The number of databases available for metabolomics continues to expand and has unique utility depending on the research question. A more expansive discussion of metabolomics database resources is available (Go, 2010). To facilitate large-scale exposomic studies, the field may benefit from having database search functionalities specifically dedicated to environmental exposures. As discussed above, discovery experiments are typically most successful when a small subset of relevant features can be targeted for structural identification and follow-up hypotheses. Thus, databases and repositories that curate information on the human exposome (e.g., the HEALS GeoDB) could provide powerful mechanisms for prioritizing features of interest to environmental health scientists.

### Bioinformatic challenges

Exposomics requires extensive bioinformatics for data reduction and analysis (Manrai et al., 2017). The NIEHS Exposome Workshop (Dennis et al., 2017) covered bioinformatic needs that are specific to exposomic biomonitoring approaches, seeking to link internal biochemical perturbations to external exposures. “*Through pathway analysis and data extraction algorithms, biological pathway perturbations can provide great insight into broad disease processes. Additionally, detection of low-level xenobiotic and unknown chemicals can be greatly enhanced through bioinformatic techniques. Further development of bioinformatic tools and data storage and handling will be key to advancing our understanding of the health impact of complex exposures*.” The large numbers of signals in omics measurements compromise the identification of effect signatures and may lead to over-fitted associations with exposure characteristics. This calls for a reduction in data dimensionality through mechanistic reasoning and biomarker identification (Blaauboer et al., 2012) to discriminate relevant signals from epi-phenomena and random responses (Hartung et al., 2012) (see Section 9 below).

Since the exposome can involve various types of data, such as biological, economic, behavioral, and social data, there will be challenges to model data from these areas and from individual, group and ecologic levels in regards to their role as determinants of disease. The key element of analysis will be the pattern recognition in the exposome of people with different exposures or disease manifestations. Pattern recognition is a particular strength of machine learning.

Machine learning comes in different flavors (Jason Brownlee’s Machine Learning Mastery blog lists 14 types^15^). The most important forms are supervised learning (we first categorize the data, e.g., exposed/not exposed or healthy/diseased), unsupervised learning (the computer generates clusters based on correlations, etc.), and reinforcement learning (a class of problems where an agent must learn to operate in an environment using feedback without a fixed training dataset, rather a goal to achieve, actions they may perform, and feedback about performance toward the goal) (Sarigiannis et al., 2018c). A broader discussion goes beyond the scope of this article, but it is obvious that these technologies lend themselves to the mining of exposome datasets.

In light of the exposome’s data-driven approach, analytic plans will need to accommodate the complex and high-dimensional nature of environmental mixtures that reflect large to small biomolecules or biomarkers (e.g., proteome and metabolome), while recognizing their interrelatedness in model specification and statistical analysis. As exposomic analyses generate specific hypotheses about cause and effect, analytic techniques will ultimately need to include multiple testing, false discovery thresholds, joint modeling of exposures and health outcomes across the lifespan (Li et al., 2019), paucity of data on multiple time scales due to the varied nature of exposures, and the need for replication to validate initial associations (Sarigiannis et al., 2018c).

### The quality assurance challenge

Exposomics requires carefully collected and well-maintained biospecimens. This includes the methods of specimen collection and storage, as well as study design and available metadata. Quality assurance (QA) continues with the data-generating technologies employed and, in the case of metabolomics, this is in its infancy (Bouhifd et al., 2015b; Beger et al., 2019). An example from our own work is Myint et al. (2017), showing joint bounding of peaks across samples improving the differential analysis in MS-based metabolomics. The QA of data processing has been mentioned above. We will later argue that mechanistic *in vitro* models might enhance the mechanistic understanding and thereby corroborate identified patterns and clusters of findings. This means that the QA aspects of *in vitro* work (Hartung, 2007; Pamies and Hartung, 2017; Pamies et al., 2017, 2018) and their reporting (Hartung et al., 2019; Krebs et al., 2019) apply also to exposomics. Ultimately, Good Exposome Practices (GEP) will have to be defined to allow assessment of study quality.

### The chemical mixture challenge

**Fig. 2:**
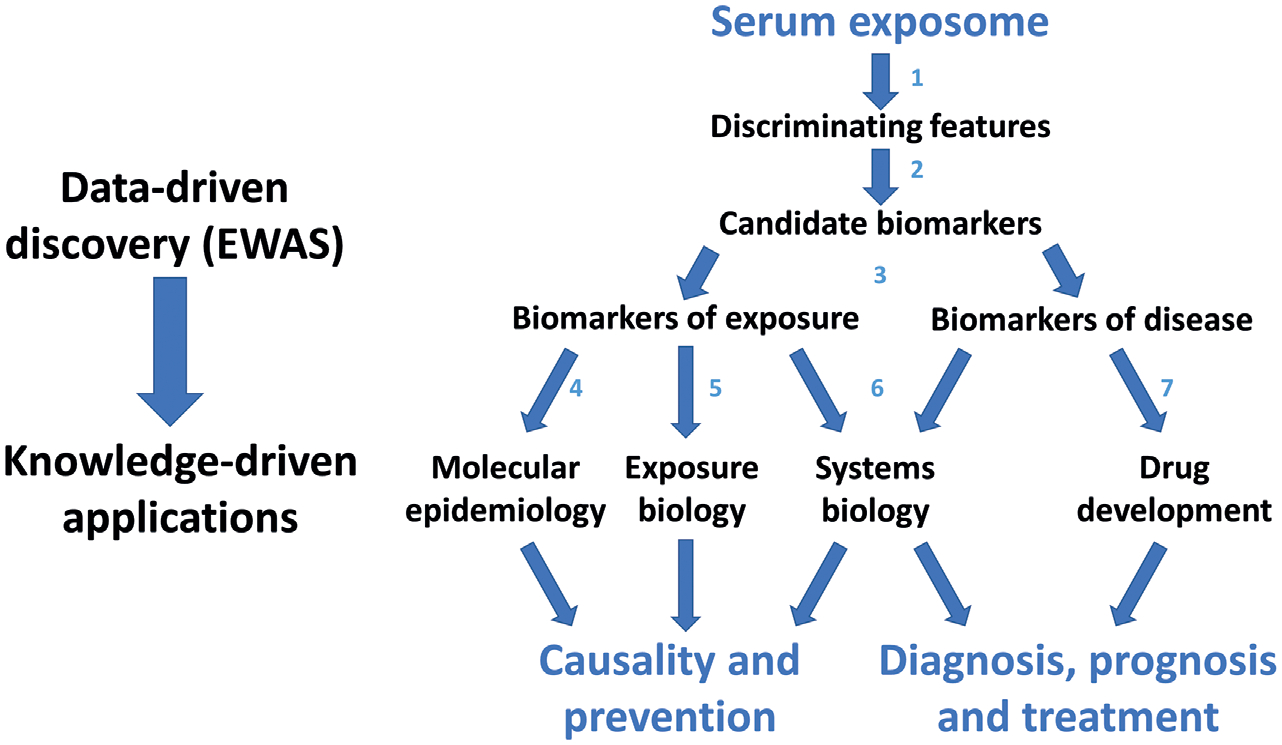
Scheme for conducting exposome-wide association studies (EWAS) to discover serum biomarkers of exposure and disease and for applying biomarkers to investigate disease causality, prevention, diagnosis and treatment Redrawn from Rappaport (2012). (1) Diseased versus healthy (case control studies), *untargeted designs*; (2) chemical identification; (3) dose-response; (4) diseased vs. healthy (prospective cohorts), *targeted designs*; (5) identify sources and measure exposures; (6) genomics, epigenetics, transcriptomics & experiments; (7) disease stage and response to therapy.

Environmental factors act in concert, changing phenotype and disease risks. The sheer number of possible combinations of exposures makes the identification of mixture-phenotype associations an analytic challenge. According to Patel (2017), “*The total number of sets of exposures that can exist is immense… For example, if our study had measured three (e.g., serum lead, cadmium, and arsenic) exposures in total, the number of possible combinations … is 3, including (lead, cadmium), (lead, arsenic), and (arsenic, cadmium). Suppose we measure 100 exposures (N = 100): the total number of combinations of size two that could co-occur is 4950. Of size 5, the total combinations are on the order of millions (exactly 75,287,520). One can imagine the scale of the number of combinations when scaling up to 100s of exposures of the exposome. Therefore, a primary challenge of identification of mixtures in phenotypes is the expansive number of exposure combinations possible*.” Identifying mixture effects is resource-intensive and requires big data (i.e., sample sizes and power). Afterwards, the verification of synergistic relationships is not as straightforward as for single exposure-phenotype associations, including (Vrijheid, 2014): (1) the evaluation of risk estimates for many single exposures in an agnostic, hypotheses-generating manner (EWAS, i.e., exposome-wide association study) (Fig. 2); (2) the evaluation of risk estimates for combined exposures through data-driven dimension reduction methods; and (3) the evaluation of risk estimates for groups of subjects sharing a similar exposome, such as in a Bayesian profile regression analysis.

## 9 The exposome meets the toxome – mechanistic toxicology to help understand exposome patterns

In the end, the patterns associated with exposures and effects need to make biological sense. They must reflect the bodies’ response. Therefore, the idea that mechanistic understanding can support or even validate (Hartung et al., 2013) findings is very attractive. Our mechanistic knowledge was previously poorly organized in the scientific literature. Independently, Hartung and McBride (2011) and Ankley et al. (2010, 2016) suggested formalized frameworks, termed pathways of toxicity (PoT) (Kleensang et al., 2014) and adverse outcome pathways (AOP), respectively. The former gave rise to the Human Toxome Project (Bouhifd et al., 2015a), while the latter led to much broader activities, including at the Organisation for Economic Co-operation and Development (OECD) level, which marginalized the human toxome approach.

**Fig.3:**
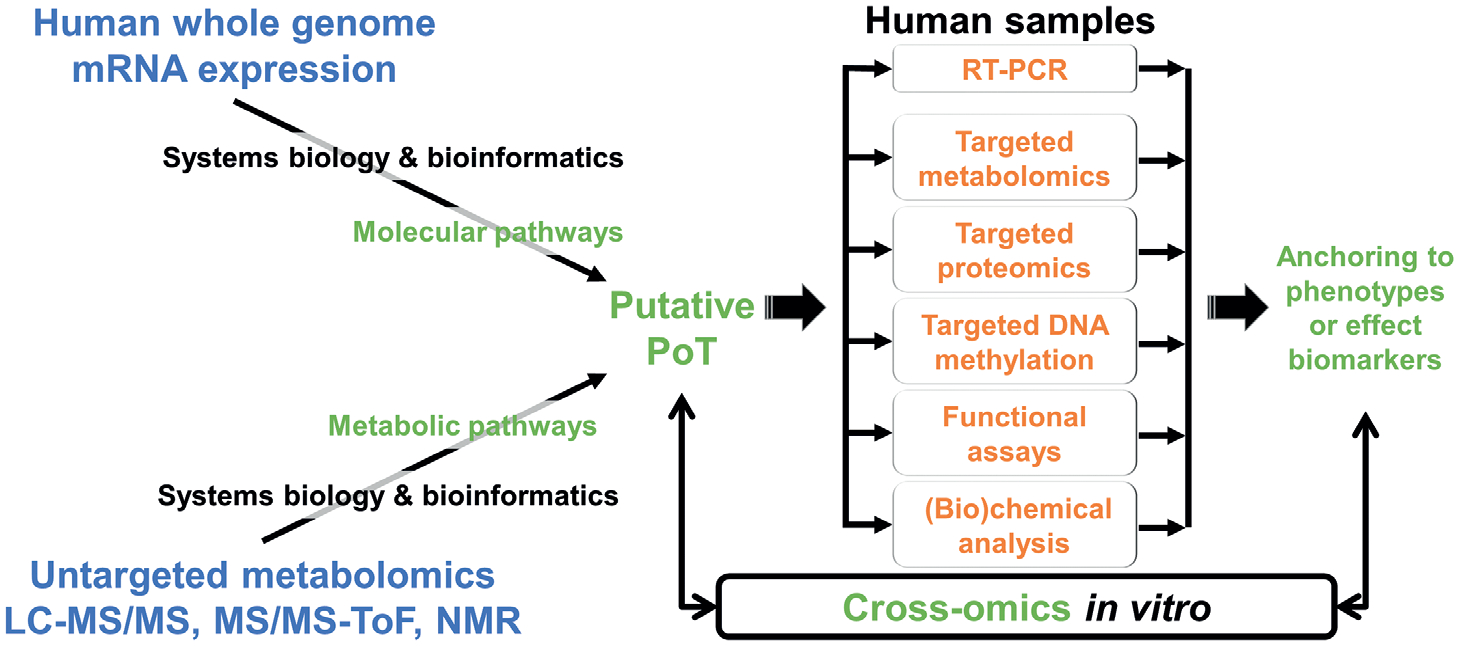
The interlink of mechanistic toxicology and the exposome Mostly untargeted technologies (blue) are used to broadly characterize the exposome and then deduce a putative pathway of toxicity (PoT). Using targeted approaches (orange), this is validated to anchor to adversity. The elements typical for a PoT/AOP approach (green) interlink these.

Some conceptual differences are important for the exposome approach: While AOP essentially organize existing knowledge to reduce biases and to organize complex chains of events from exposure to adversity, the PoT employ multi-omics approaches from *in vitro* models to postulate quantitative networks of events. Although based on different criteria (Hartung et al., 2012), these two approaches establish a framework and serve as a gateway for systems toxicology (Sturla et al., 2014; Sauer et al., 2015; Hartung et al., 2017). More generally, while AOP are ideally structured for regulatory toxicology and integrated testing strategies, PoT – with their emphasis on a network-level description of molecular events – are more tailored for understanding the complexity of exposomics, which involves multiple chemicals with multiple targets over a broad timespan. Figure 3 shows how an untargeted exposome can lead to a PoT hypothesis, which then allows its targeted evaluation.

The topic of combining AOP/PoT with the exposome was addressed in a workshop organized by the Integrated Project EXPOSOME at the UFZ Helmholtz Centre for Environmental Research in Leipzig, Germany, in 2015 (Escher et al., 2017). The workshop identified the consideration of endogenous and non-chemical stressors as well as mixture effects through which the exposome enriches the AOP concept. AOPs could further help to anchor system-wide responses to dominant modes or mechanisms of action, and thus increase the confidence in a hypothesis by providing mechanistic plausibility. Consideration of individual susceptibility to exposures is another element of the exposome that enriches the toxome. In this context, susceptibility is affected by genetics, prior exposure pattern, and age of exposure. Aggregate exposure pathways (AEPs) (Teeguarden et al., 2016) have been put forward as a concept to relate external exposures to internal actions on the receptor (i.e., sites of interaction with the body) and triggering the molecular initiating event (MIE) of the AOP. To further explore the utility and benefit of future application of AOPs in exposome assessment, the Leipzig workshop recommended (Escher et al., 2017) strengthening the following research topics:

1. “More AOPs should be developed and deposited in central databases (e.g., https://aopwiki.org/). With every new AOP developed the capacity of exposome assessment based on biological responses will increase significantly.
2. Evolutionarily conserved cellular toxicity pathways may serve as common denominators for integrated effect assessments. We advocate the use of [key events] KEs across species for a systems biology-assisted approach to exposome assessment.
3. Chronic exposure (both in terms of exposure duration and delay of effects) represents the typical environmental situation and is most relevant for human health. AOPs for chronic toxicity are at present not well-described. … an AOP-based exposome assessment should target chronic endpoints and disease outcomes.
4. The research question is an important driver of the type of exposome/AOP research, such as the identification of the main chemical risk drivers in relation to mode of action, the complex multifactorial influences of mixtures or identification of a threshold level that can explain adverse effects.”

## 10 Combining the exposome and predictive (*in silico*) toxicology tools

Computational toxicology (Hartung and Hoffmann, 2009) is fueled by new approaches, growing computational power, and curated databases (Patlewicz and Fitzpatrick, 2016). The description of an application of big data and AI in toxicology by Luechtefeld et al. (2018b) is an example from our own research which illustrates the enormous computational effort. The approach, developed in collaboration with Underwriters Laboratories (UL), a safety standard and testing organization, and by creating the spin-off, ToxTrack LLC^16^ (Hartung, 2016; Luechtefeld and Hartung, 2017; Luechtefeld et al., 2018a), was to organize similar chemicals to derive a prediction, similar to a read-across – a RASAR (read-across-based structure/activity relationship). Briefly, the approach combined several reliable data sources (PubChem^17^, ECHA (Luechtefeld et al., 2016), Integrated Chemical Environment (ICE)^18^…) and used the most common Chemical Similarity (PubChem2D) and Tanimoto (Jaccard) metrics. Network features using proximity to positive and negative neighbors and data fusion making use of other toxicity, biological and physicochemical endpoints (not only the property to be predicted) were employed. In total, 74 properties were included, which means that each chemical was characterized by a 222-dimensional vector (74 for the chemical itself and 74 each for the closest negative and positive neighbor). Machine learning (logistic regression, random forest) results in probabilistic hazard estimates using computing clusters (Apache Spark pipeline), allowing massive scale computing. It took a 180-core Amazon cloud server two days to calculate the similarity map for the 10 million structures in our database at about $5,000 in computing costs. More than 10 trillion one-to-one comparisons were made. The resulting map has chemicals that are similar to each other close together, and those less similar more distant from each other. Now, any chemical, whether included in the original 10 million structures or not, can be placed into the map with “only” half a billion operations performed in less than one second using a standard computer.

To evaluate the quality of predictions, all chemicals with a known classification were predicted in a five-fold cross-validation, which means that 190,000 predictions were made, pretending there was no information on the substance and then comparing the prediction to the known result. This was done for all chemicals, even where no close or contradictory information on a chemical’s neighbors was available. A remarkable 87% correct results were obtained for nine common toxicological hazards.

In parallel, the original ECHA database was scrutinized for chemicals for which multiple animal test results were available. 350–750 chemicals with repeat tests were found for each of the six most commonly used toxicity tests. They were, on average, 81% reproducible. However, this number is inflated by the non-toxic substances, which typically remain negative in retests. Only 69% reproducibility was found for toxic chemicals. The UL Cheminformatics Suite^19^ has been available since December 2018. The expanded database now contains 30 million structures, 300,000+ with biological and 60,000+ with animal data. The FDA has announced the formal evaluation of the tool for cosmetic ingredients and food additives.

It is now a most promising exercise to combine this approach with exposure information such as toxicants identified in exposomics studies. At this stage, this is limited by uncertainty in feature identification and the fact that the most relevant chronic and systemic toxicities have not yet been implemented.

## 11 What is in the box for alternatives to animal testing?

The current risk paradigm is essentially based on a hazard and an exposure assessment. Owing to the complexity of exposure assessment, with the notable exception of drugs with defined doses, the emphasis is typically placed on hazard. Some legislation is even *entirely* hazard-based, such as European biocides and plant-protection-product legislation or the 1958 US Delaney clause (which banned carcinogenic substances from being added to food^20^).

Hazard is traditionally assessed by animal testing. Placing stronger emphasis on exposure, or anticipating it before or concomitant to hazard assessment, gives opportunities to tailor and shortcut animal testing. An example is the concept of the threshold of toxicological concern (TTC) (Hartung, 2017), applicable where exposure assessment shows that expected doses are far below levels likely to produce a health effect. Hartung and Leist (2008), to the best of our knowledge, first suggested an internal TTC, where only the bioavailable fraction (e.g., blood levels) is used to establish whether a critical concentration is reached, “*The idea might be carried further by identifying internal TTCs, i.e. threshold peak plasma levels of toxicological concern, by measuring actual bio-availability. Increasingly, it is recognised how valuable toxicokinetic information for chemicals would be in order to carry out risk assessments and especially to integrate* in vitro *and* in vivo *data*.”

Partosch et al. (2015), Coecke et al. (2013), as well as Hartung (2017) have developed the argument for an internal TTC further. The concept gained some traction with a push from the cosmetics industry (Ellison et al., 2019). Here, we clearly overlap with the blood level determinations of toxicants and their metabolites in the exposome. Substances not measured at relevant levels in blood might be weeded out or “put on the back-burner” for hazard assessment, saving unnecessary animal testing. Notably, this is, in the context of the exposome, only possible for substances that are in broad use – typically existing high-production volume chemicals or where data from human trials can be produced. The leverage of an exposure-driven testing approach (Rowbotham and Gibson, 2011; Sewell et al., 2017), i.e., tailoring testing needs to actual exposure, is obvious.

A noteworthy example is the work carried out in HEALS, where environmentally relevant doses were used for the assessment of neurodevelopmental perturbations caused by co-exposure to phthalates and metals based on *in vitro* assays (Sarigiannis et al., 2018a) and human cohort data. HepaRG cells were exposed to mixtures of DEHP, DiNP, and BBzP phthalates, lead, and mercury. These were the most abundant pollutants in the REPRO PL (Polańska et al., 2011) and PHIME (Trdin et al., 2019) cohorts that studied environmental causation of neurodevelopmental disorders in neonates and children across Europe. The effective concentrations of the chemicals *in vitro* were estimated by extrapolation from human biomonitoring data through internal dosimetry modeling using the INTEGRA computational platform (Sarigiannis et al., 2016). Cross-omics analyses were performed on the treated cell models, including transcriptomics, proteomics, and metabolomics. Cross-omics data were analyzed using bioinformatics algorithms involving normalization, quality control, advanced statistics, and cross-omics pathway analysis with GeneSpring GX V.14.9. Integrated pathway-level analysis of transcriptomics and proteomics data revealed that co-exposure to phthalates and heavy metals leads to perturbation of the urea cycle due to alterations in the expression levels of arginase-1 and -2, argininosuccinate synthase, carbamoyl-phosphate synthase, ornithine carbamoyltransferase, and argininosuccinate lyase. Co-mapping of proteomics and metabolomics data revealed that their common drivers are responsible for the homeostasis of metabolic pathways related to choline, phosphatidylcholine, phospholipases, and triacylglycerol metabolism. The identification of the urea, phosphatidylcholine biosynthesis I and phospholipase metabolic pathways is of particular interest since these pathways also have been identified in human samples from the REPRO PL and PHIME cohorts using untargeted metabolomics analysis and have been associated with impaired psychomotor development in children from the age of three to six. The importance of the study becomes evident in the capacity of the *in vitro* system to identify the key pathway perturbations associated with these adverse outcomes in humans. This example paves the way for successful prediction of human adverse outcomes using *in vitro* models treated with doses that represent real-life exposure levels, highlighting the importance of the exposome concept in predictive toxicology. Thus, the combination with mechanistic *in vitro* models might enhance the mechanistic understanding and thereby corroborate identified patterns and clusters of findings.

## 12 Outlook and conclusions

Albert Einstein (1879 −1955) once said, “*We cannot solve our problems with the same thinking we used when we created them*.”^21^ The exposome is such an out-of-the-box approach, as it gives up the single chemical/single hazard paradigm, which has explained some public health problems but has perhaps also misdirected us by focusing on a few but not necessarily the most important ones. Juarez (2019) summarized the enormous potential of the exposome in public health: “*The exposome provides a systems science approach to bringing together and organizing data needed to model the relationships, mechanisms, and pathways among and between external exposures, endogenous exposures, health outcomes, and population-level health disparities. It holds promise for identifying completed exposure pathways from source of exposure in the natural, built, social, and policy environments to route of entry into the body, biomarkers of exposure*, *biomarkers of disease, disease phenotype, clinical outcomes, and population level disparities, across the lifespan, and between generations*.”

As laid out above, the exposome paradigm brings together exposure science advances, mechanistic toxicology, molecular epidemiology, and bioinformatics/big data analytics. It promises to improve approaches in some key challenges for toxicology and public health, namely:

- Co-exposures at low doses
- Chronic/long-term exposures at low doses
- The role of (epi)genetics and age-related susceptibility in toxicological responses
- Understanding intersecting pathways of toxicity
- Improved public health protection via precision prevention

Dennis et al. (2017) emphasized the rigor and expense for traditional biomonitoring method development, which translates into a slow and costly process for new chemicals of interest. They also state that such analyses often require relatively high volumes of sample, “*Typically 0.5–1 mL for a single method (~ 10 mL urine and > 20 mL serum to measure the 250–300 currently biomonitored chemicals), which can be limiting for certain biospecimen types and age groups under study. Yet targeted analyses are valuable given the accuracy and depth at which a chemical of interest can be assessed. By coupling traditional biomonitoring methods with broader exposomic approaches, the benefits of both strategies can be fully realized… Through untargeted biomonitoring approaches such as high-resolution metabolomics (HRM), > 1,500 metabolites can be monitored with a relatively small amount of biological specimen (≤ 100 μL) and for the cost of a single traditional biomonitoring analysis of 8–10 target chemicals…To understand the complexity of exposures faced throughout the lifespan, both traditional and nontraditional biomonitoring methods should be used. Through hybrid approaches and the integration of emerging techniques, biomonitoring strategies can be maximized in research to define the exposome*.”

Another interesting opportunity lies in the utility of exposome studies in vulnerable populations: Smith et al. (2015) have proposed using exposomics tools to quantify cumulative risk and suggested engaging affected communities in participatory exposome research. Potential populations of interest include migrant populations, newborn infants, and communities located near multiple sources of pollution.

Dennis et al. (2017) also emphasized the “fit-for-purpose” concept, which addresses the balance between overall cost of analysis and the degree of analytical rigor required to use the internal exposure measure results for a given purpose. “*In instances where legal implications exist or regulatory decisions are to be made, maximum analytical rigor is required. But for exploratory studies and for many epidemiologic studies, statistical power derived from a larger number of samples, but with sufficient precision to detect differences, is often preferred. In these cases, relaxation of analytical rigor may translate into lower costs that, in turn, could enable the number of samples analyzed to increase. Furthermore, in untargeted approaches, authentic standards are not always necessary to evaluate a chemical’s relationship to disease or alterations in biomolecular concentrations. In addition, many ‘add-on’ studies use samples collected for different analyses for which the sample collection/storage may represent more imprecision, thus not warranting the increased cost of strict analytical rigor*.” Box 1 shows further recommendations from the NIEHS workshop:

As Buck Louis and Sundaram (2012) summarized, “*Moving forward requires greater trans-disciplinary awareness of the exposome paradigm, a willingness for novel thinking and exploration including recognition of the need to measure both the external and internal environments… and identifying suitable resources for both proof-of-concept or other types of observational research*.” The agnostic approach of the exposome, however, can be problematic for grant funding, which still favors hypothesis-driven research.

A number of international activities are driving the development of the exposome concept and its implementation. On the US side, the NIEHS has been a driving force of exposomics^22^, making it part of both the 2012–2017 and 2018–2023 NIEHS Strategic Plans. In 2013 and 2018, NIEHS funded the Health and Exposome Research Center: Understanding Lifetime Exposures (HERCULES) at Emory University^23^, which is conducting exposome-focused research and also developing new tools and technologies for assessing the exposome. In 2015, NIEHS launched the Children’s Health Exposure Analysis Resource (CHEAR), a major new infrastructure to provide researchers access to laboratory analyses of biological samples from NIH-funded studies on children’s health, which has supported more than 30 studies. In 2019, NIEHS started an expanded program, called the Human Health Exposure Analysis Resource (HHEAR)^24^, which provides a larger community of researchers access to centralized, high-quality exposure assessment services. The ultimate goal of understanding the qualitative and quantitative relationships between exposures and their socio-demographic determinants and the profiles determined by the omics techniques is pursued by the establishment of the Johns Hopkins Exposome Collaborative (Box 2). The challenge lies in the quality of the data, and integrated analysis and statistical modeling of all the big data sets generated, which is why we propose to combine the exposome with AI (Hartung et al., 2019). We propose to establish a functional framework, allowing for full integration of genomics with high-quality exposomics data (GxE), by leading the field towards cutting-edge standardized methodologies and approaches, best practices (Good Exposome Practices, GEP) and ethical standards, community participation, and AI-driven big data analyses to better address public health disparities. Across the Atlantic, the European Commission established the European Exposome Cluster. The aim of this cluster, which ran from 2012 until 2019, was to create a framework for the exposome. Three projects were part of this cluster, i.e., EXPOsOMICS^25^, HELIX^26^ and HEALS^27^. The CROME-LIFE project^28^ ran from 2013 to 2018, focusing on applications of the exposome in the environment and health problems in the Mediterranean region. In addition, the NEUROSOME^29^ integrated training network, running from 2017 to 2021 with European and American participation, promises to unravel the neurological exposome, focusing on exposome-based investigation of environmental factors causing or contributing to neurodevelopmental and neurodegenerative disease. An upcoming EU Human Exposome Project will put €100 million into the exposome; its launch event has been announced^30^. In Japan, an Exposome Conference was held (Zhang et al., 2019) and the Japan Environment and Children’s Study (JECS) identifies harmful factors in the environment that affect children’s growth and health from birth to 13 years of age (2011–2032) with some exposomics aspects.

The Global Exposome Harmonization Project was recently initiated between US universities including Columbia, Mt. Sinai, Emory and Mayo Clinic with INSERM (France), Masaryk University (Czech Republic), Utrecht University (The Netherlands), University of Antwerp (Belgium), the Helmholtz Society (Germany), University of Vienna (Austria), and Imperial College London (UK). It aims at validation (inter-/intra-laboratory), harmonization of exposome measures and standardization of operating procedures, radical transparency, shared pooled standards and shared bioinformatic platforms. This promises to be a major advance for the field, but little information is currently publicly available.

The biomarker concept offers great promise in refining exposure assessment, establishing the biological plausibility of an exposure-disease association, and evaluating intervention studies. Biomarker techniques, such as the omics approaches, have shown promise and can provide information on mode-of-action and dose-response relationships (Thomas et al., 2011). As these techniques evolve, estimation of internal dose and response markers will be a critical test of the new technologies for application in risk assessment strategies (DeBord et al., 2015).

Improved exposure assessments can feed into the systems biology approach to evaluate how exposures disrupt normal biological processes. Systems toxicology is the integration of classical toxicology with quantitative analysis of large networks of molecular and functional changes occurring across multiple levels of biological organization (Hartung et al., 2012, 2017; Sturla et al., 2014). The connectivity theory (Schmutz et al., 2004) proposes a new environmental health impact assessment paradigm, addressing the complexity related to the interplay of genetic, epigenetic, environmental, dietary, and sociodemographic parameters. This requires coupling high-dimensional biological analysis and system science using big data analytics and bioinformatics. Connectivity applied on the exposome elucidates toxicity pathways (Rappaport, 2018; Vrijheid et al., 2014; Sarigiannis, 2019a) and unravels causal associations between environmental stressors and health, paving the way towards precise prevention and targeted policy interventions (Turner et al., 2018). Determining prevalent co-exposures and related main effects (Sarigiannis and Karakitsios, 2018) using machine-learning methods and data analytic approaches (“exposome-wide association studies”, i.e., EWAS) (Fig. 2) on large cohorts is expected to enhance our capacity to identify the most harmful environmental chemical mixtures (Sarigiannis, 2019b). The feasibility of using mechanistic analysis of toxicity pathways using multi-omics data (Turner et al., 2018) has been demonstrated, for example, in the NIH Human Toxome Project (Bouhifd et al., 2015a; Kleensang et al., 2014). Analysis of downstream metabolic products or altered gene expression could provide the entry points for precision medicine (Hartung and McBride, 2011), while the integration of genome and exposome data can support precise prevention, both on population and individual levels (Sarigiannis et al., 2018b).

At the Johns Hopkins Exposome Collaborative launch, Denis Sarigiannis ended his presentation with the following remarks: “*Exposome science can overhaul the current environmental health risk assessment paradigm. This requires the combination of:*

- Refined external and internal exposure assessment
- High-dimensional biology and system science aiming at integration using big data analytics (multi-omics) and bioinformatics
- Deeper integration is needed regarding human and in vitro data; there is higher convergence as we move to pathways that regulate homeostasis.
- Cross-omics analysis and interpretation of metabolic pathways allow us to target our interventions against the mechanism that is highly perturbed – ‘pathway activation’ based interventions.

*This has implications on both community and personalized levels: At community level, the most cost-efficient policies should be prioritized (exposome informed policy-making) and at individual level, targeted dietary and lifestyle recommendations should be derived, for minimizing exposure, as well as the propagation of the adverse outcome pathways.”*

On the same occasion, Gary W. Miller concluded by saying, “*For years the exposome has been a non-descript, fuzzy, and, at times, rather unscientific idea. Today, we can say that the exposome represents a new science – a new way of approaching how the environment influences health. Truly a new discipline. As such, the field must establish and defend its identity, develop its core principles (i.e. the constellation), and forge a path forward (journal, society, regular meetings, websites, etc.)*.”

We are witnessing a scientific revolution in the making in toxicology (Hartung, 2008).

## Box 1: Recommendations NIEHS Workshop 2015

(Dennis et al., 2017)

1. Encourage secondary analyses of samples collected for traditional targeted chemical studies.
2. Evaluate and use standardized measurement platforms with measurement harmonization.
3. Use existing resources and databases to obtain information on current exposures that may be important.
4. Provide guidance for the use of existing databases and develop tools to allow searches across multiple databases.
5. Foster and facilitate discussion with people from different disciplines to discuss the reality of targeted and untargeted analytic capabilities.
6. Develop chemistry methods to enable the detection of low-abundance chemicals and to enable differentiation of endogenous molecules from exogenous molecules.
7. Develop bioinformatics techniques to enhance detection of unknown chemicals using untargeted methods.
8. Encourage development of pharmacokinetic models.

## Box 2: The Exposome Collaborative

### @ Johns Hopkins University

The Exposome Collaborative at Johns Hopkins University was funded in January 2019. Its purpose is to congregate the intellectual and material resources housed under the various disciplines within environmental health sciences and engineering and to evaluate the exposome in a holistic manner.

Goals of The JHU Exposome Collaborative:

- Bring together expertise in all the different disciplines needed to better characterize the exposome in human health studies.
- Develop the needed tools and analyses for exposome assessment.

The ultimate goal is not only to provide a functional exposome framework, but also to lead the field in, e.g., establishing best-practices, ethics standards, standardized methodologies, and multi-modal data analyses using artificial intelligence. Public health studies implementing the established exposome framework will have a competitive advantage over other studies that, e.g., solely focus on genomics or traditional exposure assessments, as the exposome framework will agnostically be able to capture yet unexplained causes of health disparities and identify desperately needed targets for intervention.

The JHU Exposome Collaborative was kicked-off with a launch event on November 8, 2019, to which the authors of this article contributed. Seed funding was provided through the generous donation of Krieger School of Arts & Sciences alumnus Yu Wu (MS ’89, PhD ’96) and Whiting School of Engineering alumna Chaomei Chen (MS ’88) to the Department of Environmental Health and Engineering.

#### Steering Committee & Principal Investigators:

*Founders:* Gurumurthy Ramachandran, PhD and Thomas Hartung, MD PhD

*Director:* Fenna Sillé, PhD

*Associate Director:* Alexandra Maertens, PhD

*Members:* Carsten Prasse, PhD, Kirsten Koehler, PhD, Ana Rule, PhD, Andre Kleensang, PhD, Hugh Ellis, PhD, Keeve Nachman, PhD, Scot Miller, PhD, Lesliam Quiros-Alcala, PhD, Meredith McCormack, MD

*Contact:* exposome@jhu.edu

## Footnotes

1
https://ehe.jhu.edu/research/the-exposome-collaborative/

2 Sandra Bullock Quotes. BrainyQuote.com, BrainyMedia Inc, 2019. https://www.brainyquote.com/quotes/sandra_bullock_579786 (accessed 28.12.2019).

3 Dan Quayle Quotes. BrainyQuote.com, BrainyMedia Inc, 2019. https://www.brainyquote.com/quotes/dan_quayle_387491 (accessed 28.12.2019).

4
http://www.itmonline.org/arts/pulse.htm

5 Robert Orben Quotes. BrainyQuote.com, BrainyMedia Inc, 2019. https://www.brainyquote.com/quotes/robert_orben_104953 (accessed 28.12.2019).

6
http://geneontology.org

7
https://www.brainyquote.com/quotes/luther_burbank_277483

8
https://oshwiki.eu/wiki/Biological_monitoring_(biomonitoring)#Advantages_and_disadvantages_of_biological_monitoring

9
https://www.cdc.gov/nchs/nhanes/index.htm

10
https://apps.who.int/iris/bitstream/handle/10665/39037/9241571551-eng.pdf?sequence=1&isAllowed=y

11
https://www.ncbi.nlm.nih.gov/books/NBK326791/

12 Introduction to a series of twelve workshops at the Fields Institute for Research in the Mathematical Sciences

13
https://metlin.scripps.edu/landing_page.php?pgcontent=mainPage

14
http://www.hmdb.ca

15
https://machinelearningmastery.com/types-of-learning-in-machine-learning/

16
https://toxtrack.com

17
https://pubchem.ncbi.nlm.nih.gov

18
https://bit.ly/2QrAg15

19
https://msc.ul.com/en/products/cheminformatics/

20
https://en.wikipedia.org/wiki/Food_Additives_Amendment_of_1958

21
https://www.brainyquote.com/quotes/albert_einstein_121993

22
https://www.niehs.nih.gov/research/supported/exposure/bio/index.cfm

23
http://emoryhercules.com/

24
https://hhearprogram.org

25
http://www.exposomicsproject.eu/

26
http://www.projecthelix.eu/

27
http://www.heals-eu.eu/

28
https://www.crome-life.eu/

29
https://www.neurosome.eu/

30
https://europa.eu/newsroom/events/launch-event-european-human-exposome-network_en

## Glossary

(Dennis et al., 2017; DeBord et al., 2016; and other sources)

Aggregate exposure pathways (AEPs): The AEP concept has been developed complementary to the AOP describing the key events from source via external exposure (including environmental and dietary exposure) to exposure in the organism, termed target site exposure.
Biomarker: A characteristic that can be objectively measured and evaluated as an indicator of normal biological processes, pathogenic processes or pharmacological responses to a therapeutic intervention
Biomonitoring: The measurement of the body burden of toxic chemical compounds, elements, or their metabolites, in biological substances
Children’s Health Exposure Analysis Resource (CHEAR): NIEHS project to advance understanding about how the environment impacts children’s health and development (2015-)
Eco-exposome: The extension of exposure science from the point of contact between a stressor and receptor inward into the organism and outward to the general environment, including the ecosphere
Epigenetics: The study of changes in organisms caused by modification of gene expression rather than alteration of the genetic code itself
Epigenetic scar: Long-term changes in epigenetics induced by stressors, which affect regulation of gene expression
Exposome: Concept describing the totality of exposure experienced by an individual during their life and the health impact of those exposures (Wild, 2005), redefined (Miller and Jones, 2014): The cumulative measure of environmental influences and associated biological responses throughout the lifespan, including exposures from the environment, diet, behavior, and endogenous processes.
EXPOsOMICS: European-funded project under the FP7 Exposome Program that aims to develop a new approach to assessing environmental exposures in adults, particularly through the use of omics techniques
Environment- or exposome-wide assessment studies (EWAS): Studies that collect multiple kinds of exposure data from multiple sources, which are then related to health effects
Feature: Raw data output from mass spectrometry analysis, which includes an accurate mass-to-charge ratio with associated retention time (RT) and ion intensity. A feature can represent one or more chemicals/metabolites, so data extraction methods are critical to interpretation
Geographic Information Systems (GIS): Systems that manage geographic data
HELIX (The Human Early-Life Exposome): European-funded project under the FP7 Exposome Program focused on understanding the early-life exposome through novel exposure measurement and data-driven methods
HERCULES (Health and Exposome Research Center: Understanding Lifetime Exposures): US NIEHS – funded center at Emory University focused on providing infrastructure and expertise to develop and refine new tools and technologies to advance exposome research as well as on promoting environmental health sciences research overall
HEALS (Health and Environment-wide Associations based on Large population Surveys): A European Commission-funded project focused on integrating omics data and traditional biomonitoring measurements with alterations in outcomes such as gene expression and metabolic regulation to assess environmental exposures and human health associations
HHEAR (Human Health Exposure Analysis Resource): US NIEHS program that provides a larger community of researchers access to centralized, high-quality exposure assessment services
High-resolution metabolomics: Mass spectrometry technique that can detect > 10,000 features through instrumentation such as time-of-flight, Fourier transform ion cyclotron resonance, and orbitrap mass analyzers
International Exposome Center, the I3 Care Centre: International Exposome Center between the Netherlands, Hong Kong and Canada aiming at expanding the exposome concept through the development of new investigative tools to discover and quantify modifiable risk factors
Japan Environment and Children’s Study (JECS): Study to identify harmful factors in the environment affecting children’s growth and health, and to investigate the relationship between such factors and children’s health condition involving 100,000 parent-child pairs followed from birth to 13 years of age (2011–2032)
Multiplexing: Fractionation of samples to remove higher-level chemicals, enabling detection of lower-abundance chemicals
Metabolomics: The characterization, identification, and quantitation of metabolites resulting from a wide range of biochemical processes in living systems, typically by MS and NMR technologies.
Omics technologies: The collective characterization of components and measurement of molecules from a biological field of study, which involves a large-scale data acquisition system that can be used to measure biological states or responses; examples include the genome (DNA), transcriptome (RNA), and proteome (proteins).
Phenome: All of the phenotypes of a cell, tissue, or organism
Reality mining: Analysis of behavioral and self-reported data extracted from social networks and other portable devices’ applications
Reverse causality: A situation in which the outcome precedes and causes the exposure instead of the other way around. This is of particular concern in retrospective and cross-sectional studies.
Semi-targeted/hybrid approaches: Exploits the advantages of both targeted and untargeted approaches: for example, using metabolomics for discovery of potential exposures followed by targeted analysis for a more fully quantitative measure
Systems toxicology: Systems biology applied to toxicology, i.e., the integration of classical toxicology with quantitative analysis of large networks of molecular and functional changes occurring across multiple levels of biological organization
Testing multiple variables for associations: Statistical inference problem especially with the use of omics methodologies, which involve the simultaneous testing of thousands of null hypotheses. The multitude of comparisons made in these studies will result in both false positive (type 1 errors) and, if the correction for multiple comparisons is overly conservative or power is inadequate, false negative (type 2 errors) results.
Traditional biomonitoring/targeted analyses: Analyses of biological samples for specific chemicals: either exposures or markers of exposures
Untargeted analyses: Agnostic analyses that can measure a broad set of endogenous and exogenous metabolites in one sample run
